# Liver Fibrosis and 8-Year All-Cause Mortality Trajectories in the Aging Cohort of the Salus in Apulia Study

**DOI:** 10.3390/biomedicines9111617

**Published:** 2021-11-04

**Authors:** Roberta Zupo, Fabio Castellana, Sara De Nucci, Giovanni De Pergola, Madia Lozupone, Ilaria Bortone, Marco Castellana, Giancarlo Sborgia, Luisa Lampignano, Gianluigi Giannelli, Francesco Panza, Rodolfo Sardone

**Affiliations:** 1 Unit of Data Sciences and Technology Innovation for Population Health, National Institute of Gastroenterology “Saverio de Bellis”, Research Hospital, Castellana Grotte, 70013 Bari, Italy; zuporoberta@gmail.com (R.Z.); fabio.castellana@irccsdebellis.it (F.C.); sara.denucci@irccsdebellis.it (S.D.N.); madia.lozupone@gmail.com (M.L.); ilariabortone@gmail.com (I.B.); mcastellana01@yahoo.it (M.C.); luisalampignano@gmail.com (L.L.); f_panza@hotmail.com (F.P.); 2 Unit of Geriatrics and Internal Medicine, National Institute of Gastroenterology “Saverio de Bellis”, Research Hospital, Castellana Grotte, 70013 Bari, Italy; gdepergola@libero.it; 3 Department of Basic Medical Sciences, Neuroscience and Sense Organs, University of Bari “Aldo Moro”, 70124 Bari, Italy; gcsborgia@hotmail.it; 4 Scientific Direction, National Institute of Gastroenterology “Saverio de Bellis”, Research Hospital, Castellana Grotte, 70124 Bari, Italy; gianluigi.giannelli@irccsdebellis.it

**Keywords:** chronic liver disease, frailty, survival, biomarkers, liver fibrosis

## Abstract

Age is a major contributor to the liver fibrosis rate and its adverse health-related outcomes, including mortality, but older populations are still under-explored. We investigated multimorbidity and inflammatory biomarkers in relation to the increasing liver fibrosis risk to delineate 8-year all-cause mortality trajectories in 1929 older adults from the population-based Salus in Apulia Study. Liver fibrosis risk was assumed using the fibrosis-4 (FIB-4) score, assigned to three liver fibrosis risk groups (low, intermediate, high). In the secondary analyses, the APRI score was also calculated to allow for comparisons. Male subjects (prevalence difference: −13.49, 95% confidence interval (CI): −18.96 to −8.03), a higher multimorbidity burden (effect size, ES: −0.14, 95% CI: −0.26 to −0.02), a higher prevalence of physical frailty (ES: 6.77, 95% CI: 0.07 to 13.47), and a more pronounced inflammatory pattern as indicated by tumor growth factor-α circulating levels (ES: −0.12, 95% CI: −0.23 to −0.01) were significantly more common in the highest-risk FIB-4 score group. Liver function characterized by lipid profile and platelet levels worsened with increasing FIB-4 risk score. The 8-year risk of death was nearly double in subjects in the highest-risk FIB-4 score group, even after controlling for possible confounders. Furthermore, a steeper mortality curve was clearly observed for FIB-4 scores as compared with the APRI scoring system with respect to liver fibrosis risk. In conclusion, using a scoring tool based on simple routine biomarkers to detect liver fibrosis risk may enhance biological knowledge of age-related outcomes of chronic liver disease and be helpful in the clinical setting to identify subjects at risk for adverse health-related outcomes, including mortality.

## 1. Introduction

The demographic shift is spotlighting the exponential growth in aging populations worldwide. Population growth projections over the next decade are worrying, and there is some doubt as to whether we will be able to satisfy the health demands of almost 9 billion people [1]. From a generational point of view, the older age population will contribute significantly to the healthcare demand, as this part of the population places a significant burden on general well-being and quality of life [2]. Indeed, although the quality of aging is benefiting from accumulating knowledge and experience [3], it still involves a ruinous continuum of decline in multiple functional physiological domains.

Biological phenomena underlying the aging trajectories are still debated. Several cellular and molecular events have been postulated to develop malfunction throughout late-life, leading to phenotypic patterns of chronic disease accumulation in older age, including neurodegenerative disorders, cardiovascular disease, diabetes, and cancer. A recent report from the Global Burden of Disease Study suggests that only 4% of the world population is disease-free, and multimorbidity, described as the most common chronic condition, affects almost half of the population aged over 65 [4]. From a global perspective, Western countries face increasing epidemic rates of multimorbidity, likely due to substantial changes in lifestyle over the past decades.

Chronic liver disease (CLD) constitutes a spectrum of conditions carrying a heavy public health burden. Currently, around 2 million deaths per year worldwide are caused by CLD, 1 million being due to cirrhosis and 1 million to viral hepatitis and hepatocellular carcinoma (HCC). Cirrhosis is currently the 11th most common cause of death globally [5]. However, while the prevalence of viral hepatitis is expected to decrease due to the availability of highly potent direct-acting antiviral drugs, alcohol consumption and poor lifestyle remain the leading cause of CLD [6]. Today, non-alcoholic fatty liver disease (NAFLD) stands out as the most common liver disease worldwide, and it is not surprising that the prevalence of obesity, dyslipidemia, metabolic syndrome, and diabetes mellitus are concomitantly and steadily increasing and that the prevalence tends to accumulate during aging.

Although the natural history of CLD encompasses progression to cirrhosis and HCC, not all affected subjects undergo this progression. It seems that liver fibrosis is the main determinant of disease progression; indeed, people with a higher degree of liver fibrosis are more prone to poorer long-term outcomes [7,8]. The histological spectrum of NAFLD ranges from simple steatosis, usually considered rather benign, to non-alcoholic steatohepatitis (NASH), characterized by lobular inflammation. Patients with NASH are more likely to progress to advanced fibrosis, cirrhosis, and eventually to HCC [9].

Timely and proper management of CLD staging is key to stratifying patients risk to fit effective healthcare strategies, in order to cut healthcare costs and improve quality of life in older age, that is often compromised in CLD [10]. As such, alternative approaches to liver biopsy for liver fibrosis screening are advocated; of these, the most widely used to date are based on noninvasive risk assessment tools, i.e., imaging methods and combined scores of clinical and serum indicators. The fibrosis-4 (FIB-4) score [11] is extensively used as a surrogate predictive model for the screening of liver health in the general population, and recently a cutoff to detect the probability of observing fibrosis was validated on a population aged 65+ [7].

Across large population-based studies, this scoring system has shown a good predictive ability for general, cardiovascular, and specific-cause mortality, both when restricted to individuals with NAFLD [11] and applied to the general population. Specifically, there are reports linking this scoring system to specific death from SARS-CoV-2 and incident heart failure, as well as to liver disease itself [12,13]. Moreover, data on older people are very limited. In this context, very recent findings from the InCHIANTI prospective study provided evidence that the fibrosis risk in late-life was closely associated with a raised hazard for general and cardiovascular mortality and physical disability, regardless of multimorbidity and other potential confounders [14]. We assessed the large dataset of the population-based Salus in Apulia Study to evaluate multimorbidity and inflammatory biomarkers, as well as other routine biomarkers, in relation to three risk categories of the noninvasive liver fibrosis score (FIB-4) and 8-year all-cause mortality among older adults in Southern Italy.

## 2. Methods

### 2.1. Study Population

Participants of the present study were recruited from the electoral rolls of Castellana Grotte (Apulia, Italy). The sampling framework was the health registry office list until 31 December 2014, which included 19,675 subjects, of which 4021 were aged 65+ years [15]. All of the participants were part of the “Salus in Apulia Study”, a public health initiative run by the National Institute of Gastroenterology IRCCS “Saverio De Bellis” Research Hospital and supported by the Italian Ministry of Health and the Regional Government of Apulia. The mortality data were obtained from the electronic health records of the Regione Puglia, updated until 31 May 2020. This study employed data from a subset of the Salus in Apulia Study, which included 1929 older people who completed all of the evaluations [15]. The study was authorized by the IRB of the head institution, the National Institute of Gastroenterology and Research Hospital “Saverio de Bellis” in Castellana Grotte, and all subjects provided informed permission prior to their evaluation (Apulia, Southern Italy) [15]. The study met the principles of the Helsinki Declaration and adhered to the “Standards for Reporting Diagnostic Accuracy Studies” (STARD) guidelines (http://www.stard-statement.org/, accessed on 10 September 2021) and the “Strengthening the Reporting of Observational Studies in Epidemiology” (STROBE) guidelines.

### 2.2. Clinical and Laboratory Examination

Years of schooling were used to define education. After overnight fasting, a blood sample was taken in the morning to determine the levels of fasting blood glucose (FBG), glycated hemoglobin (HbA1c), total cholesterol, high-density lipoprotein (HDL) cholesterol, low-density lipoprotein (LDL) cholesterol, and triglycerides using standard automated enzymatic colorimetric methods (AutoMate 2550, Beckmann Coulter, Brea, CA, USA) under strict quality control [15].The Friedewald equation was used to determine LDL cholesterol. The glucose oxidase technique was used to determine plasma glucose (Sclavus, Siena, Italy). A Coulter Hematology analyzer (Beckman–Coulter, Brea, CA, USA) was used to determine the blood cell count [15]. Extemporaneous ambulatory systolic and diastolic blood pressure were measured using the OMRON M6 automated blood pressure monitor in a sitting posture after at least a 10 min rest at least three times throughout the clinical examination. Automatic enzyme techniques were used to assess alanine amino transferase (ALT), aspartate amino transferase (AST), and gamma glutamyl transferase (GGT). Serum insulin concentrations were determined using a radioimmunoassay (Behring, Scoppito, Italy) and serum 25(OH) vitamin D concentrations were determined using a chemiluminescence technique (Diasorin Inc., Stillwater, OK, USA); all samples were examined twice. A latex particle-enhanced immunoturbidimetric assay (Kamiya Biomedical Company, Seattle, WA, USA) was used to measure serum high-sensitivity C-reactive protein (CRP) (reference range: 0–5.5 mg/L; interassay coefficient of variation: 4.5%). The ELISA quantitative sandwich enzyme approach was used to measure serum interleukin (IL)-6 and tumor growth factor-alpha (TNF-α) (QuantiKine High Sensitivity Kit, R&D Systems, Minneapolis, MN, USA and QuantiGlo immunoassay from R&D Systems, Minneapolis, MN, USA). The interassay coefficients of variation for IL-6 were 11.7% and 13.0%, respectively. In the same facility, the findings of the inflammatory marker tests were analyzed under stringent quality control techniques.

### 2.3. Multimorbidity, Non-Communicable Diseases, and Cardiovascular Risk Score

The presence of two or more chronic diseases at the baseline examination, i.e., diabetes mellitus, hypertension, peripheral age-related hearing loss, vision loss, cognitive impairment, asthma, chronic obstructive pulmonary disease, and late-life depression, was defined as multimorbidity status, i.e., multimorbidity score, as described in detail elsewhere [15,16]. The ASCVD (atherosclerotic cardiovascular disease) risk score was calculated in accordance with the national guideline developed by the American College of Cardiology (ACC) as an estimate of the 10-year risk of having a cardiovascular problem, such as a heart attack or stroke [17].

### 2.4. Anthropometric Assessment

A skilled nutritionist carried out the clinical procedures (RZ). All anthropometric measurements were taken while the individuals were dressed in light clothing and were not wearing shoes. After an overnight fast, all variables were collected at the same time between 7:00 and 10:00 a.m. A wall-mounted stadiometer was used to measure height to the nearest 0.5 cm (Seca 711; Seca, Hamburg, Germany). A calibrated balance beam scale was used to determine body weight to the closest 0.1 kg (Seca 711; Seca, Hamburg, Germany). Body mass index (BMI) was computed by dividing body weight (kg) by the square of height (m^2^) and categorized using WHO standards [18]. The narrowest section of the abdomen, or the area between the tenth rib and the iliac crest, was used to estimate waist circumference (minimum circumference).

### 2.5. Assessment of Physical Frailty and Liver Frailty

Assessment of the physical frailty status was performed using the operational CHS criteria—that is, positivity to three or more of the following components: weight loss, exhaustion, low levels of physical activity, weakness, and slow movement, as detailed elsewhere [2]. Subjects who met three or more criteria were included in the frailty group; all the others were classified as non-frail subjects.

### 2.6. Alcohol Intake Assessment

Dietary habits of the previous year, assessed by a self-administered Food Frequency Questionnaire, as described in detail elsewhere [19], were extracted from the Salus in Apulia dataset and used to derive data on alcohol consumption of all participants. Estimates of daily alcohol consumption were derived from Italian food composition tables [20]. According to American and European norms for daily alcohol consumption, a threshold of 20 g/day in females and 30 g/day in males was used [21].

### 2.7. Non-Invasive Liver Fibrosis Assessment

A liver fibrosis score was calculated according to the FIB-4 equation including age, AST, ALT, and platelets [11]; we used age-specific cut-points for subjects aged 65+ years, as suggested by McPherson and colleagues [22], for assessing the risk of liver fibrosis. Accordingly, patients were assigned to 3 groups: low-risk (score < 2.0), intermediate-risk (2.0 < score < 2.67), and high-risk of advanced fibrosis (score > 2.67). We chose to apply this new intermediate cutoff for subjects aged ≥65 years while maintaining the cutoff for the highest risk group, given its proven effectiveness in improving specificity for advanced fibrosis, effectively controlling the false positive rate, and avoiding an unfavorable increase in the false-negative rate of the test. In the secondary analysis, the APRI scoring was also calculated according to the previously referred cutoffs, i.e., APRI < 0.5 to identify a fibrosis-free liver, APRI > 0.5 for liver fibrosis and APRI > 1.5 for probable cirrhosis [23].

### 2.8. Statistical Analysis

The whole sample was subdivided according to the three risk categories of FIB-4 scoring (low-risk or “fibrosis excluded” if <2.0, intermediate-risk or “needs further investigation” if ranging between 2.0 and 2.67, and high-risk or “fibrosis likely” if above 2.67) to describe clinical and functional differences in terms of frequency and associations between those groups. Normal distributions of quantitative variables were tested using the Kolmogorov-Smirnov test. Because of the normal distribution of the variables, data are reported as mean ± SD for continuous measures and frequency and percentages (%) for all categorical variables. Differences in the prevalence exposure groups (FIB-4 categories), and other categorical variables and their 95% confidence intervals (CI) were calculated and used to assess important practical differences in the magnitude of association, i.e., effect size (ES) [24]. Differences between continuous variables were calculated using Cohen’s d difference between means and Glass’ delta when the assumption of similar variance was violated, and their ES using confidence intervals around them. To study the time between entering the study and a subsequent event, the non-parametric Kaplan–Meier method was used to explore survival probability, and the log-rank test was applied to evaluate the equality of survival among categories. Three nested Cox multivariable models were used to estimate the hazard ratio (HR) of mortality for the primary factors (intermediate and high FIB-4, and range values indicative of liver fibrosis and probable cirrhosis for the APRI) because they also allow assessment of the survival HR for an individual, given a prognostic variable (measured continuously or categorically). The Cox proportional hazard model was fitted to the data, and the proportional hazard assumption was evaluated by means of Schoenfeld residuals (SRT). All fitting models were assessed using Akaike Information Criteria (AIC) and the Bayesian information criterion (BIC). Risk estimators are expressed as HR and 95% CI. The multicollinearity of models was evaluated through the variance inflation factor (VIF), using the score of 2 as a cutoff for exclusion. Confounders were selected among those factors retained related to general mortality, such as age, sex, smoking habits, education, alcohol consumption, and multimorbidity [25] for the Cox models.

## 3. Results

In the population examined (N = 1929), males were slightly predominant (50.5%). Mean age was 73.56 ± 6.30 years. Table 1 summarizes the differences among the baseline sociodemographic and clinical characteristics of the study population according to the liver risk score group. The frequency of low, intermediate, and high FIB-4 scores across groups was 58.10% (N = 1120), 19.4% (N = 374), and 22.6% (N = 435), respectively. The prevalence difference (PD) between sexes was proportionally higher for males from the low- to high-risk group. Male subjects were significantly more common in the high than low FIB-4 score group (PD: −13.49, 95% CI: −18.96 to −8.03) and in the intermediate than low FIB-4 score group (PD: −10.79, 95% CI: −16.61 to −4.98). The mean age, and magnitude of its effect, was proportionally higher across each increasing FIB-4 score group in the transition from the low-risk group to the intermediate-risk group (ES: −0.44, 95% CI: −0.56 to −0.32) and from the intermediate-risk group to the high-risk group (ES: −0.34, 95% CI: −0.48 to −0.20).

Education level was significantly lower among subjects in the high-risk fibrosis group (ES: 0.23, 95% CI: 0.11 to 0.34). The same group had a higher burden of multimorbidity on average (ES: −0.14, 95% CI: −0.26 to −0.02 from the mid to high FIB-4 score), a more pronounced inflammatory profile, as indicated in low vs. high FIB-4 score groups by average circulating TNF-α levels (ES: −0.12, 95% CI: −0.23 to −0.01), IL-6 (ES: −0.12, 95% CI: −0.23 to −0.01), as well as a both higher physical frailty prevalence difference (ES: 6.77, 95% CI: 0.07 to 13.47) and 10-year CV risk, as assessed by the ASCVD scoring system and indicated in comparing low vs. high FIB-4 score groups (ES: −0.18, 95% CI: −0.29 to −0.07). No meaningful difference was observed for BMI, smoking habits, and systolic blood pressure across groups. Furthermore, circulating 25-hydroxyvitamin D3, FBG, and HbA1c did not change significantly across the three groups.

Lipid profile followed a trend of consistency according to worsening of the liver condition. Particularly, lipids were lower, on average, in the high-risk FIB-4 score group compared with the lower-risk and intermediate-risk groups (ES: 0.37, 95% CI: 0.26 to 0.48 and ES: 0.21, 95% CI: 0.07 to 0.34 for total cholesterol levels; ES: 0.31, 95% CI: 0.19 to 0.42 and ES: 0.15, 95% CI: 0.01 to 0.28 for LDL cholesterol levels, and ES: 0.18, 95% CI: 0.07 to 0.30 and ES: 0.16, 95% CI: 0.02 to 0.30 for triglyceride levels, respectively). The same rationale likely drives the lowering of mean insulin levels across groups (ES: 0.21, 95% CI: 0.09 to 0.33 and ES: 0.24, 95% CI: 0.13 to 0.35, respectively, from the low-risk group to the intermediate-risk group and from the intermediate-risk group to the high-risk group).

**Figure 1:**
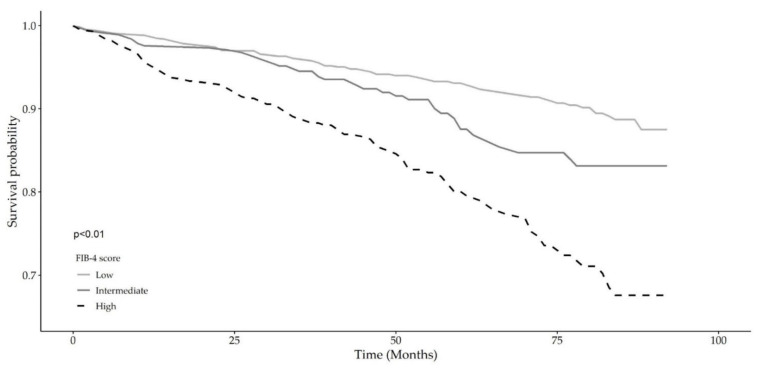
Kaplan–Meier survival probability curves for the three categories of fibrosis-4 (FIB-4) score (low-, intermediate-, and high-risk).

**Figure 2:**
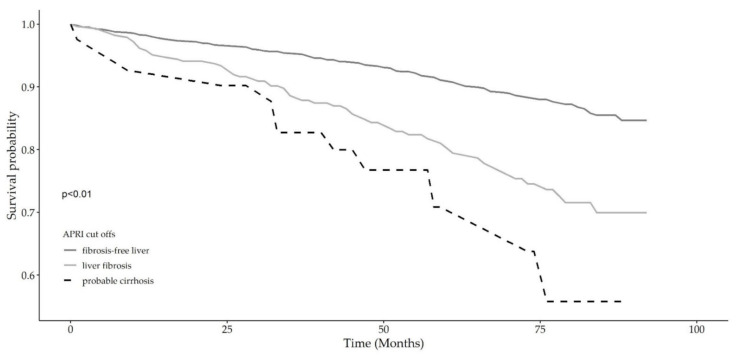
Kaplan–Meier survival curves for the probable fibrosis according to APRI scores: fibrosis-free (APRI < 0.50), liver fibrosis (APRI < 1.50) and probable cirrhosis (APRI ≥ 1.50).

Table 2 shows the results of nested multivariable Cox regression models on the liver fibrosis risk categories expressed by the FIB-4 (low-, intermediate-, and high-risk) and APRI (fibrosis-free (APRI < 0.50), liver fibrosis (APRI < 1.50) and probable cirrhosis (APRI ≥ 1.50)) scoring systems, hierarchically adjusted for selected confounding factors, i.e., model 1: unadjusted, model 2: age and sex, model 3: age, sex, alcohol consumption, education, smoking habits, and multimorbidity. The FIB-4 risk score was shown to retain significance even after full adjustment that included multimorbidity as a continuous variable (HR: 1.80, 95% CI: 1.31 to 2.47). Similarly, the APRI score also showed significance in the three models even after full adjustment (HR: 2.18, 95% CI: 1.19 to 3.98), but from a comparative perspective, FIB-4 showed better risk prediction. Combined Kaplan–Meier survival probability analyses across liver fibrosis risk categories for both scores (Figure 1 and Figure 2) showed a strong significant association with overall mortality over 8 years (92 months). From a comparative liver fibrosis risk perspective, high FIB-4 scores (high fibrosis risk) were shown to be more predictive of overall death than high liver fibrosis risk as assessed by the APRI score (HR: 1.80, 95% CI: 1.31 to 2.47 and HR: 1.69, 95% CI: 1.24 to 2.30, respectively). Kaplan–Meier survival probability curves for the three fibrosis-4 (FIB-4) score categories (low-, intermediate-, and high-risk) and for the three APRI score categories ((fibrosis-free (APRI < 0.50), liver fibrosis (APRI < 1.50) and probable cirrhosis (APRI ≥ 1.50)) are shown in Figure 1 and Figure 2, respectively. A steeper curve is clearly observed for FIB-4 scores compared with APRI with respect to liver fibrosis risk.

## 4. Discussion

The present study offered evidence that older adults with a high risk of liver fibrosis according to the noninvasive FIB-4 scoring system have poorer 8-year survival, and yet this scoring system performs better in comparison with the APRI in terms of predicting overall mortality. This relationship was consistent also after adjustment for all selected possible confounders, including multimorbidity, suggesting that the FIB-4 score may predict all-cause mortality independent of the presence of other major and coexisting chronic diseases. The present finding is expected to be of interest within clinical screening contexts and comprehensive geriatric assessment (CGA), suggesting new applicative scenarios of the FIB-4 well beyond single-use for prognostic liver fibrosis purposes, covering a broader spectrum in predicting liver health trajectories and major health-related outcomes.

The rationale for using the FIB-4 was the extent of its validation as a non-invasive and straightforward scoring method, useful in screening for liver impairment and fibrosis [10], much more predictive over our outcome than APRI probably due to the inclusion of age as a variable in the score calculation too. This kind of tool has proven validity to assess liver fibrosis in subjects affected by any liver disease, including NAFLD, chronic hepatitis C virus (HCV) infection, and HIV/HCV co-infection [26]. Higher FIB-4 scores have been associated with all-cause mortality in several chronic diseases, such as microscopic polyangiitis, rheumatoid arthritis, and heart failure [27,28,29]. However, the proportion of older subjects included in previous studies was negligible, with the notable exception of another population-based study with a shorter follow-up period suggesting that older persons classified by non-invasive scores as having higher liver fibrosis risk were also at increased risk for mortality and incident disability [14]. The present findings indicate that scoring a cluster of purely liver-related variables works well in skimming the older age population for hazard trajectories, supporting the concept that liver-related factors may play, along with other risk factors that accumulate with aging, an important and independent role in the pathophysiological patterns underlying the occurrence of adverse health-related outcomes.

In line with this hypothesis, subjects falling into the high-risk group for liver fibrosis had higher circulating levels of inflammatory biomarkers such as TNF-α. In nonalcoholic cirrhosis as an outcome of the progression of NASH, changes in clinical parameters (indicating the development of hepatocellular deficiency, altered protein and lipid metabolism, progressive inflammation) are accompanied by specific changes in levels of biochemical and molecular-genetic indicators of apoptosis and inflammation [30,31]. In the present study, liver function as indicated by lipid profile and platelet levels worsened with increasing risk score for FIB-4. In fact, some markers of liver function such as platelets and white and red blood cells are a common hematological complication of CLD. Thrombocytopenia, which is frequently observed in patients with CLD and cirrhosis, can manifest due to a decreased thrombopoietin production and accelerated platelet destruction caused by hypersplenism [32]. In addition, the mean serum values of LDL cholesterol, HDL cholesterol, total cholesterol, and triglycerides decreased significantly with increasing CLD severity. In fact, with increasing hepatic parenchymal damage, there was a decrease in these lipid parameters [33], given the impaired liver synthesis function in these patients. Low plasma levels of triglycerides, free fatty acids, total cholesterol, HDL cholesterol, lipoprotein(a), apolipoprotein A-I and A-II, and apolipoprotein B were also observed in HCC [33]; this may be due to hepatocellular impairment, also suggesting a poor prognosis.

In support of the internal validity of the present findings, an increased risk of liver fibrosis was observed in males and subjects with less schooling. Epidemiological metrics reported that males are twice as likely to die of CLD and cirrhosis as females [34]. As a possible proposed causal pathway, adult females face major changes in their hormonal status, driven by a decrease in estrogen levels, which appears to play a protective role for the liver, particularly against both the progression of HCV-related fibrosis and the occurrence of HCC [35,36]. However, the explorative window in this area is still broad. Furthermore, a clearer link has been recently described between educational level and CLD, suggesting an independent association linking education with viruses and alcohol-related etiology [36]. Different non-invasive methods for risk stratification, i.e., FIB-4, the NAFLD Fibrosis Score (NFS), and the AST/platelet ratio index (APRI) have limited performance in predicting changes in fibrosis, as evaluated by future biopsies, but they consistently demonstrated the ability to predict liver-related morbidity and mortality, with a level of performance that met or exceeded that of a liver biopsy [37].

Some potential limitations need to be considered when interpreting our findings. First, mortality was not attributed to a specific disease, and thus we could not analyze the association of the liver disease score with cause-specific death. Second, although factors related to the specific etiology of CLD (i.e., alcohol consumption or virus exposure) may influence the observed association with all-cause mortality, we were able to keep track limited to our data availability. Thus, we fully-adjusted our analyses for alcohol use, reaching no significant changes in risk estimates. Additionally, we had no information on markers of viral hepatitis. However, the prevalence of viral hepatitis among our rural older age population in Southern Italy is reported to be around 7% [38], and thus could likely have had a limited impact on our results. Moreover, information on liver fibrosis status, as detected by transient elastography (FibroScan), was not available. To address this limitation, we used the FIB-4 score as a surrogate, as this provides a more accessible screening tool for physicians, especially general practitioners. However, the lack of any polypharmacy mention contributes to limit the completeness of our data. The strengths of this study included its long-term prospective observation time (92 months of follow-up), the large population-based sample size, and the generalizability of the results to the southern Mediterranean population.

Looking ahead to a multidimensional CGA screening of older adults for the risk of adverse health-related outcomes, taking advantage of simple diagnostic algorithms to predict these events may be the best way to act early. The use of such scores, based on simple routine biomarkers for the detection of liver fibrosis, could both improve biological knowledge of age-related outcomes of CLD and be used in clinical settings to identify older individuals at risk for adverse health-related outcomes, regardless of the presence of other accumulated chronic diseases. Last but not least, the easy-to-use feature unlocks a window for use even by non-medical healthcare professionals.

## Figures and Tables

**Table 1:** Baseline sociodemographic and clinical characteristics of the study population according to fibrosis-4 (FIB-4) score. The Salus in Apulia Study (N = 1929).

	FIB-4 Score			Effect Size ^‡^		
	Low (a)	Intermediate (b)	High (c)	(a) vs. (b)	(a) vs. (c)	(b) vs. (c)
Proportions (%)	1120 (58.10)	374 (19.40)	435 (22.60)			
Observation time (months)	55.61 ± 22.32	55.93 ± 21.49	55.79 ± 22.51	−0.01 (−0.13 to 0.10)	−0.01 (−0.11 to 0.10)	−0.01 (−0.13 to 0.14)
*Sociodemographic variables*						
Age (years)	71.98 ± 5.71	74.56 ± 5.99	76.77 ± 6.61	−0.44 (−0.56 to −0.32)	−0.80 (−0.91 to −0.68)	−0.34 (−0.48 to −0.20)
Sex						
Males	508 (45.40)	210 (56.10)	256 (58.90)	**−10.79 (−16.61 to −4.98)**	**−13.49 (−18.96 to −8.03)**	−2.70 (−9.53 to 4.13)
Females	612 (54.60)	164 (43.90)	179 (41.10)			
BMI (kg/m^2^)	28.47 ± 4.85	28.2 ± 4.74	28.55 ± 5.08	0.05 (−0.06 to 0.17)	−0.01 (−0.12 to 0.09)	−0.07 (−0.21 to 0.06)
Education (years)	7.19 ± 3.85	6.9 ± 3.97	6.32 ± 3.57	0.07 (−0.04 to 0.19)	**0.23 (0.11 to 0.34)**	**0.15 (0.01 to 0.29)**
Smoking habits (%)	97 (8.70)	27 (7.20)	27 (6.20)	−1.44 (−4.54 to 1.66)	−2.45 (−5.26 to 0.35)	**0.16 (0.02 to 0.30)**
SBP (mmHg)	132.7 ± 14.27	133.26 ± 14.71	133.79 ± 14.87	−0.03 (−0.15 to 0.07)	−0.07 (−0.18 to 0.03)	−0.03 (−0.17 to 0.12)
DBP (mmHg)	78.55 ± 7.54	77.91 ± 7.84	77.03 ± 8.86	0.08 (−0.03 to 0.20)	0.17 (0.06 to 0.28) *	0.10 (−0.04 to 0.24) *
*Blood biomarkers*						
FBG (mg/dL)	105.1 ± 27.33	107.33 ± 31.9	106.54 ± 27.7	−0.07 (−0.19 to 0.05) *	0.02 (−0.11 to 0.16) *	0.03 (−0.11 to 0.17) *
HbA1c (mmol/mol)	40.18 ± 9.91	41.15 ± 12.41	40.76 ± 10.24	−0.08 (−0.20 to 0.04) *	−0.05 (−0.16 to 0.05) *	0.03 (−0.10 to 0.17) *
Insulin (μU/mL)	9.51 ± 7.02	8.34 ± 5.56	8.03 ± 6.14	0.21 (0.09 to 0.33) *	0.24 (0.13 to 0.35) *	0.05 (−0.09 to 0.19) *
TC (mg/dL)	187.93 ± 37.28	181.83 ± 37.2	174.2 ± 35.29	**0.16 (0.04 to 0.28)**	**0.37 (0.26 to 0.48)**	**0.21 (0.07 to 0.34)**
HDL C (mg/dL)	49.3 ± 12.94	48.25 ± 13.4	47.21 ± 12.81	0.08 (−0.03 to 0.19)	**0.16 (0.05 to 0.27)**	0.07 (−0.05 to 0.21)
LDL C (mg/dL)	115.79 ± 31.3	110.86 ± 32.19	106.27 ± 28.93	**0.15 (0.03 to 0.27)**	**0.31 (0.19 to 0.42)**	**0.15 (0.01 to 0.28)**
Triglycerides (mg/dL)	109.61 ± 62.48	106.85 ± 56.9	97.88 ± 60.66	0.04 (−0.07 to 0.16)	**0.18 (0.07 to 0.30)**	**0.16 (0.02 to 0.30)**
25(OH)D3 (ng/mL)	38.65 ± 17.85	39.65 ± 17.68	39.55 ± 17.37	−0.05 (−0.17 to 0.06)	−0.05 (−0.16 to 0.06)	0.01 (−0.13 to 0.14)
RBC (10^6^ cells/mm^3^)	4.83 ± 1.19	4.78 ± 0.49	4.7 ± 0.56	0.10 (−0.02 to 0.21) *	0.24 (0.12 to 0.35) *	0.15 (0.01 to 0.29) *
*Inflammatory profile*						
CRP (mg/L)	0.6 ± 0.93	0.54 ± 0.74	0.59 ± 0.74	0.09 (−0.03 to 0.21) *	0.02 (−0.09 to 0.13) *	−0.06 (−0.20 to 0.06) *
Interleukin-6 (pg/mL)	3.62 ± 6.36	4.18 ± 6.11	4.57 ± 7.99	−0.09 (−0.21 to 0.03) *	−0.12 (−0.23 to −0.01) *	−0.05 (−0.19 to 0.08) *
TNF-α (pg/mL)	2.62 ± 2.98	2.96 ± 4.28	3.17 ± 4.52	−0.08 (−0.20 to 0.04) *	−0.12 (−0.23 to −0.01) *	−0.04 (−0.18 to 0.08) *
WBC (10^3^ cells/mm^3^)	6.3 ± 1.89	6.04 ± 1.82	5.76 ± 1.77	**0.13 (0.02 to 0.25)**	**0.28 (0.17 to 0.40)**	**0.15 (0.01 to 0.29)**
*Liver Metabolism*						
FIB-4 Score	1.43 ± 0.35	2.27 ± 0.18	4.83 ± 3.2	−4.60 (−4.80 to−4.40) *	−1.06 (−1.18 to −0.94) *	−0.80 (−0.94, −0.66) *
APRI Score	0.24 ± 0.08	0.36 ± 0.12	0.78 ± 0.60	−1.02 (−1.31 to −0.89) *	−0.90 (−1.17, −0.79) *	−0.70 (−0.84 to −0.55) *
Platelets (10^3^ cells/mm^3^)	244.72 ± 57.75	201.85 ± 44.15	184.09 ± 53.58	**0.78 (0.66 to 0.78)**	**1.07 (0.95 to 1.18)**	**0.35 (0.21 to 0.49)**
AST (U/L)	22.46 ± 6.86	29.46 ± 14.64	57.17 ± 46.12	−0.48 (−0.60 to −0.36) *	−0.75 (−0.87 to −0.64) *	−0.60 (−0.74 to −0.46) *
ALT (U/L)	25.54 ± 18.93	24.39 ± 18.59	27.45 ± 25.32	0.06 (−0.06 to 0.18)	−0.08 (−0.19 to 0.04) *	−0.12 (−0.26 to 0.02) *
GGT (U/L)	28.94 ± 31.61	32.57 ± 34.63	46.52 ± 44.01	−0.11 (−0.22 to 0.01) *	−0.40 (−0.51 to −0.29) *	−0.40 (−0.51 to −0.29) *
*Non-communicable Diseases*						
Diabetes mellitus (%)	132 (11.80)	55 (14.70)	58 (13.30)	2.92 (−1.14 to 6.98)	1.55 (−2.16 to 5.26)	−1.37 (−6.18 to 3.43)
ASCVD risk score	14.8 ± 5.99	15.9 ± 6.10	15.9 ± 5.83	**−0.19 (−0.30 to −0.07)**	**−0.18 (−0.29 to −0.07)**	0.01 (−0.13 to 0.15)
Physical Frailty (%)	145 (7.50)	67 (17.90)	74 (17.00)	**7.66 (0.98 to 14.34)**	**6.77 (0.07 to 13.47)**	**0.94 (0.65 to 1.35)**
Peripheral ARHL (%)	211 (18.80)	89 (23.80)	125 (28.70)	**4.96 (0.07 to 9.84)**	**9.90 (5.07 to 14.73)**	4.94 (−1.12 to 11.00)
Hypertension (%)	783 (69.90)	262 (70.10)	302 (69.40)	0.14 (−5.22 to 5.51)	−0.49 (−5.58 to 4.61)	−0.63 (−6.98 to 5.72)
CI (%)	54 (4.80)	34 (9.10)	41 (9.40)	**4.27 (1.10 to 7.44)**	**4.60 (1.59 to 7.62)**	0.33 (−3.67 to 4.34)
Asthma (%)	108 (9.60)	38 (10.20)	32 (7.40)	0.52 (−3.00 to 4.03)	−2.29 (−5.29 to 0.71)	−2.80 (−6.73 to 1.12)
Vision loss (%)	40 (3.60)	12 (3.20)	20 (4.60)	−0.36 (−2.45 to 1.73)	1.03 (−1.22, 3.27)	1.39 (−1.27 to 4.05)
COPD (%)	196 (17.50)	78 (20.90)	69 (15.90)	3.36 (−1.32 to 8.04)	−1.64 (−5.73 to 2.45)	−4.99 (−10.35 to 0.37)
LLD (%)	80 (7.90)	26 (8.30)	34 (8.50)	0.40 (−3.08 to 3.88)	0.59 (−2.60 to 3.79)	0.19 (−3.91 to 4.29)
Metabolic syndrome (%)	134 (12.00)	49 (10.79)	51 (11.70)	−1.27 (−4.93 to 2.39)	−0.24 (−3.81 to 3.33)	1.03 (−3.32 to 5.38)
Multimorbidity (%)	17.93 ± 12.52	19.92 ± 13.74	19.64 ± 13.20	−0.14 (−0.26 to −0.02) *	−0.07 (−0.18 to 0.03)	0.05 (−0.08 to 0.19)

All data are shown as mean ± SD for continuous variables and as percentage (%) for proportions. Statistically significant values are formatted in bold font. ^‡^ Cohen’s d effect size where not otherwise specified, * Glass’ delta, prevalence difference for categorical variables. Legend: FIB-4 score cutoffs: Low: <2.00; Intermediate: 2.00< x <2.67; High: >2.67. APRI, AST-to-Platelet Ratio Index: fibrosis-free (APRI < 0.50), liver fibrosis (APRI < 1.50), and probable cirrhosis (APRI ≥ 1.50). BMI: body mass index; SBP: systolic blood pressure; DBP: diastolic blood pressure; FBG: fasting blood glucose; HbA1c: glycated hemoglobin; TC total cholesterol; HDL C: high-density lipoprotein cholesterol: LDL C: low-density lipoprotein cholesterol; 25(OH)D3: 25-hydroxyvitamin D3; TNF: tumor necrosis factor; RBC: red blood cells; WBC: white blood cells; AST: alanine aminotransferase; ALT: alanine transaminase; GGT: gamma glutamyltransferase; ASCVD risk score: atherosclerotic cardiovascular disease risk score; CI: cognitive impairment; ARHL: age-related hearing loss; COPD: chronic obstructive pulmonary disease; LLD: late-life depression.

**Table 2:** Cox multivariable regression nested models on each fibrosis-4 (FIB-4) score risk category (low-, intermediate-, and high-risk).

	FIB-4 Score			APRI Score	
	HR	95% CI		HR	95% CI
		Model 1			Model 1
Intermediate FIB-4 score	1.51	1.04 to 2.21	Liver Fibrosis	2.31	1.71 to 3.13
High FIB-4 score	3.01	2.24 to 4.06	Probable Cirrhosis	3.60	1.99 to 6.49
		Model 2			Model 2
Intermediate FIB-4 score	1.16	0.76 to 1.69	Liver Fibrosis	1.70	1.25 to 2.32
High FIB-4 score	1.76	1.28 to 2.41	Probable Cirrhosis	2.09	1.14 to 3.80
		Model 3			Model 3
Intermediate FIB-4 score	1.12	0.77 to 1.64	Liver Fibrosis	1.69	1.24 to 2.30
High FIB-4 score	1.80	1.31 to 2.47	Probable Cirrhosis	2.18	1.19 to 3.98

HR: hazard ratio; CI: confidence interval. Legend: FIB-4 score cutoffs: Low: <2.00; Intermediate: 2.00< x <2.67; High: >2.67. APRI, AST-to-Platelet Ratio Index: fibrosis-free (APRI < 0.50), liver fibrosis (APRI < 1.50), and probable cirrhosis (APRI ≥ 1.50). Model 1: Unadjusted. Model 2: Adjusted for age and sex. Model 3: Adjusted for age, sex, smoking habits, alcohol consumption, education, and multimorbidity.

## Data Availability

Data are available on request from the corresponding author.
